# Diet Contributes Significantly to the Body Burden of PBDEs in the General U.S. Population

**DOI:** 10.1289/ehp.0900817

**Published:** 2009-06-18

**Authors:** Alicia J. Fraser, Thomas F. Webster, Michael D. McClean

**Affiliations:** Boston University School of Public Health, Department of Environmental Health, Boston, Massachusetts, USA

**Keywords:** biomarkers, diet, exposure, NHANES, PBDEs

## Abstract

**Background:**

Exposure of the U.S. population to polybrominated diphenyl ethers (PBDEs) is thought to be via exposure to dust and diet. However, little work has been done to empirically link body burdens of these compounds to either route of exposure.

**Objectives:**

The primary goal of this research was to evaluate the dietary contribution to PBDE body burdens in the United States by linking serum levels to food intake.

**Methods:**

We used two dietary instruments—a 24-hr food recall (24FR) and a 1-year food frequency questionnaire (FFQ)—to examine food intake among participants of the 2003–2004 National Health and Nutrition Examination Survey. We regressed serum concentrations of five PBDEs (BDE congeners 28, 47, 99, 100, and 153) and their sum (∑PBDE) against diet variables while adjusting for age, sex, race/ethnicity, income, and body mass index.

**Results:**

∑PBDE serum concentrations among vegetarians were 23% (*p* = 0.006) and 27% (*p* = 0.009) lower than among omnivores for 24FR and 1-year FFQ, respectively. Serum levels of five PBDE congeners were associated with consumption of poultry fat: Low, medium, and high intake corresponded to geometric mean ∑PBDE concentrations of 40.6, 41.9, and 48.3 ng/g lipid, respectively (*p* = 0.0005). We observed similar trends for red meat fat, which were statistically significant for BDE-100 and BDE-153. No association was observed between serum PBDEs and consumption of dairy or fish. Results were similar for both dietary instruments but were more robust using 24FR.

**Conclusions:**

Intake of contaminated poultry and red meat contributes significantly to PBDE body burdens in the United States.

Polybrominated diphenyl ethers (PBDEs) are a class of brominated flame retardants that are commonly found in consumer products such as polyurethane foam, electronics, and textiles. Of the three commercial mixtures (penta-BDE, octa-BDE, and deca-BDE), only deca-BDE is still produced and sold in the United States (La Guardia et al. 2007). Nevertheless, vast quantities of penta-BDE and octa-BDE are present in consumer products that are still in use. Congeners from all three mixtures are found ubiquitously in both the indoor and outdoor environments, as well as in human blood and breast milk [U.S. Environmental Protection Agency (EPA) 2008]. Body burdens in North America are considerably higher than those found in Europe and Asia (Hites 2004). In a recent study based on the 2003–2004 National Health and Nutrition Examination Survey (NHANES), Sjödin et al. (2008) reported PBDE concentrations in the general population of the United States and examined associations with age, sex, and race/ethnicity.

Effects of PBDEs in experimental animals include endocrine disruption, neurodevelopmental and behavioral outcomes, hepatic abnormalities, and possibly cancer (Birnbaum and Staskal 2004; Darnerud 2008; McDonald 2002). Although little human epidemiology has yet been done, early studies suggest effects on male reproductive hormones (Meeker et al. 2009) and fertility (Akutsu et al. 2008), thyroid hormone homeostasis (Turyk et al. 2008), cryptorchidism (Main et al. 2007), and lower birth weight and length (Chao et al. 2007).

PBDEs have been measured in dust and air (Allen et al. 2008; Stapleton et al. 2005; Wilford et al. 2004, 2005) and various types of food, including beef, pork, poultry, fish, and dairy products (Huwe and Larsen 2005; Schecter et al. 2006b). North Americans are thought to be exposed to PBDEs primarily via inadvertent exposure to dust, with a smaller role of diet (e.g., Allen et al. 2007; Lorber 2008; Stapleton et al. 2008). Most exposure estimates use a combination of exposure factors and measurements of PBDEs in environmental samples. Unfortunately, the exposure factors for dust ingestion are particularly uncertain, especially for adults (U.S. EPA 1997). An alternative, complementary approach empirically examines air, dust, and diet as determinants of PBDE body burdens; only a few small studies of this kind have been done in the United States. Wu et al. (2007) found associations between penta-BDE congeners in breast milk of Boston-area residents and house dust concentrations, as well as with consumption of meat and dairy products. Schecter et al. (2006a) found a trend among U.S. adult vegans toward lower serum PBDEs with increasing years of abstinence from animal product intake. Modest associations have been reported between serum PBDEs and fish ingestion among consumers of sport-caught fish (Anderson et al. 2008; Morland et al. 2005).

Accordingly, the importance of diet to PBDE exposure in the general U.S. population is not well understood, and the NHANES population provides a unique opportunity to study this question. We examined the relationship between diet and serum concentrations of five PBDEs (BDE congeners 28, 47, 99, 100, and 153) in the 2003–2004 NHANES. As the first large-scale investigation of dietary contribution to PBDE body burdens in the United States, our objectives were *a* ) to determine whether vegetarians have lower PBDE body burdens than do omnivores; *b*) to determine which dietary factors (e.g., poultry, red meat, fish) are associated with serum PBDE levels in the U.S. general population; and *c*) to compare the usefulness of two different NHANES dietary instruments [24-hr food recall (24FR) and food frequency questionnaire (FFQ)] for evaluating diet as a determinant of serum PBDE levels.

## Materials and Methods

### NHANES and PBDEs

Administered by the National Center for Health Statistics of the Centers for Disease Control and Prevention (CDC), NHANES is a cross-sectional, nationally representative survey of the U.S. civilian, noninstitutionalized population. Released in 2-year groupings, the survey uses a multistage probability cluster sampling design to collect data on approximately 5,000 people/year (CDC 2008). A one-third random sample of participants ≥ 12 years of age (*n* = 2,337) was selected for serum PBDE analysis in the 2003–2004 survey. Ten PBDE congeners (BDEs 17, 28, 47, 66, 85, 99, 100, 153, 154, and 183) were measured using high-resolution gas chromatograph/mass spectroscopy. Results are presented on a serum lipid basis, with concentrations less than the limit of detection (LOD) replaced by the LOD divided by the square root of 2. Details of the analytical method were reported by Sjödin et al. (2004). In compliance with the NHANES Institutional Review Board, written informed consent was given by all participants before data collection.

### Dietary assessment

NHANES collects dietary information using two different methods: 24FR and an FFQ. The 24FR data provide detailed information on the types and amount of food consumed during the 24 hr (midnight to midnight) before the interview. Two 24FR interviews were conducted 3–10 days apart on different days of the week, one in-person and one by telephone. Each interview used a set of measuring guides (various glasses, bowls, spoons, a ruler, etc.) and an interview method called the Automated Multiple Pass Method (AMPM) [Agricultural Research Service (ARS) 2008]. The AMPM is a research-based five-step dietary recall method designed to enhance complete and accurate food recall while reducing respondent burden. It begins by asking respondents to recall as best they can all foods consumed during the previous 24 hr. Respondents are then probed for forgotten foods using a series of questions to spark memory. Additional details on data collection, including interviewer training and quality control methodology, are described elsewhere (CDC 2007). The 24FR data were collected from 1,971 of the participants for whom PBDEs were also analyzed, defining our study population.

For each of the > 3,000 individual food items reported through the 24FR, the U.S. Department of Agriculture (USDA) provides detailed nutritional content, including total fat (ARS 2006; Raper et al. 2004). Using USDA descriptions of each individual food, we categorized the fat content of each item as derived from poultry (chicken, turkey, duck, etc.), red meat (beef, pork, goat, etc.), dairy, egg, seafood, or non animal sources. If a food item contained fat from two categories (e.g., chicken and beef sausage), then the fat content of that food item was divided evenly between the two food categories. If a food item contained fat from more than two categories or from undetermined sources, then the fat content of that food item was categorized as mixed/unclear. Examples of such items included gumbo, luncheon meat (without further description), and baked goods (where it was unclear whether they contained vegetable oil or butter). We then summed fat intake from each food category for each participant and averaged it over the two 24FRs to estimate average fat intake from poultry, red meat, dairy, egg, seafood, non animal, and mixed/unclear.

The second NHANES dietary assessment method was an FFQ mailed to participants to collect information on the frequency of food consumption over the previous year. These data were available for 1,536 (78%) of our study population. NHANES reports daily frequency estimates for 216 food items as servings per day, although information on portion size was not collected and serving size is not uniform across food items. Thus, the NHANES FFQ is not meant to provide precise data for use in deriving estimates of absolute intake for either nutrients or foods, but rather to provide a broad picture of average intake over a longer period of time than the 24FR. We categorized the FFQ data into the seven food categories described above (poultry, red meat, dairy, egg, seafood, non animal, mixed/unclear). Daily servings of individual foods within each category were summed to create an estimate of average intake for each of the seven food categories for each participant.

### Statistical methods

We examined the relation ship between natural log-transformed, lipid- adjusted PBDE concentrations and dietary variables using multiple linear regression. Exponentiation of the resulting β coefficients describes the multiplicative increase in lipid-adjusted serum PBDE concentrations per unit change in predictor variables. Because the NHANES data are collected using a complex, multistage probability sampling design, we employed the appropriate clustering variables using SAS Survey procedures (SAS Statistical Software, version 9.1.3 for Windows; SAS Institute Inc., Cary, NC). As an alternative to incorporating the NHANES sampling weights, we included the variables used in determining the sample weights as covariates in all regression models. This method has been shown to enhance statistical efficiency when the primary research goal is determining associations within a sample population rather than estimating means for a more generalized population (Korn and Graubrad 1991, 1995).

All models were adjusted for age, race/ethnicity, sex, poverty index ratio (PIR), and body mass index (BMI). Race/ethnic categories were defined as non-Hispanic black, Hispanic (including Mexican Americans), and non- Hispanic white (including the 77 participants who reported other or mixed races/ethnicities). We used the continuous variables age and age^2^ in regression models to parsimoniously account for the nonlinear relationship observed previously reported for PBDEs (Sjödin et al. 2008); however, for descriptive statistics, we categorized age as 12–19, 20–39, 40–59, and ≥ 60 years to enhance interpretability and for consistency with Sjödin et al. (2008). We also examined the effects of PIR and BMI, two variables not included in the original NHANES analysis of PBDEs (Sjödin et al. 2008). The PIR is the ratio of a family’s income to their poverty threshold (U.S. Census Bureau 2008) and was categorized as below poverty level (< 1), 1–2, 2–4, and ≥ 4. The BMI data were categorized as underweight (< 18.5), normal (18.5–24.99), overweight (25–29.99), and obese (> 30). To enhance the interpretability of the regression results for use in figures, we calculated adjusted least-square geometric mean (GM) PBDE concentrations using the mean age (40 years) and averaging across all race/ethnicity, sex, PIR, and BMI categories.

We divided daily fat intakes obtained from 24FR for poultry, red meat, and dairy into tertiles such that one-third of the sample population fell into each category (low, medium, high). The fat intake data exhibited a relatively smooth distribution given that the data were generated by combining individual-specific meal data with meal-specific fat data. However, the FFQ intake data were less smooth, with tertile cut-points falling in the middle of peaks within the distribution. Accordingly, we adjusted cut points for the FFQ categories (low, medium, high) to ensure that groups of otherwise similar intake patterns were not artificially separated into different groups. The final categorization resulted in a larger proportion of the sample falling into the medium category of red meat intake and the low category of poultry intake.

For the 24FR data, we defined vegetarians as those who reported no consumption of poultry or red meat over the two 24-hr food periods (3%). According to the FFQ, only 18 individuals reported never having eaten poultry or red meat over the course of the year (1%), whereas others reported extremely low annual consumption. Because it has been estimated that 3% of U.S. adults consider themselves vegetarian (Vegetarian Resource Group 2003), we therefore defined vegetarians as those within the lowest 3% of combined daily poultry and red meat intake (≤ 0.14 servings/day or about once per week) when using the FFQ data.

We examined the relationship between PBDEs and diet using several different regression models: vegetarians and non vegetarians as defined by the 24FR or the FFQ; total daily fat intake (uncategorized by food type); tertiles of daily fat intake (categorized by food group in accordance with 24FR data); and tertiles of daily servings (categorized by food group in accordance with FFQ data). We examined PBDE serum concentrations on a congener-specific basis as well as on a total basis [sum of five congeners (∑PBDE)]. Tests of trend for categorical variables were performed by assigning each category a value equal to the median of all samples within that category. All statistical analyses were performed using SAS software, and statistical significance was determined with α= 0.05.

## Results

Measures for five congeners—BDEs 28, 47, 99, 100, and 153—exceeded the LOD in at least 60% of samples. Because missing values varied slightly by congener, the total number of subjects with PBDE data varied from 1,918 subjects up to 1,971 subjects. PBDE concentrations in serum lipid followed approximately log-normal distributions and were log-transformed in statistical analyses. All five PBDEs (all components of the penta-BDE commercial formulation) were strongly correlated with each other and with ∑PBDE [see Supplemental Material, Table 1 available online (doi:10.1289/ehp.0900817.S1 via http://dx.doi.org/)]. BDE-153, a component of both the penta-BDE and octa-BDE commercial formulations, had somewhat weaker associations with the other four congeners (*r*= 0.56–0.78).

Table 1 presents demographic characteristics for the 2,040 subjects with PBDE data, the 1,971 subjects who also had 24FR data, and the 1,536 subjects who also had FFQ data. The demographic characteristics of all three populations were quite similar. Table 1 also presents the number of observations in each category of poultry and red meat intake for both the 24FR and the FFQ data sets.

Table 2 presents GM concentrations of PBDEs stratified by sex, race/ethnicity, age, PIR, and BMI categories. BDE-47 had the highest GM concentration [23.2 ng/g lipid; 95% confidence interval (CI), 22.0–24.4], followed by BDE-153 (6.1 ng/g lipid; 95% CI, 5.8–6.5), BDE-99 (5.6 ng/g lipid; 95% CI, 5.3–5.9), BDE-100 (4.4 ng/g lipid; 95% CI, 4.2–4.6), and BDE-28 (1.3 ng/g lipid; 95% CI, 1.2–1.3). In unadjusted, univariate analyses, sex, age, PIR, and BMI were all significantly associated with ∑PBDE. Significant linear trends were also observed for ∑PBDE by age, PIR, and BMI categories. Concentrations were highest among males, those < 20 years of age, the poor, and the underweight (Table 2). Although ∑PBDE levels were not significantly different by race/ethnicity, black and Hispanic participants had higher ∑PBDE levels than did white participants in the unadjusted analysis.

Supplemental Material, Table 2 (doi: 10.1289/ehp.0900817.S1) summarizes the average daily consumption of poultry, red meat, dairy, egg, seafood, and nonanimal products as determined by 24FR and FFQ. As discussed in “Materials and Methods,” 3% of the population was classified as vege tarian using the FFQ. Results from the 24FR analysis were consistent with this estimate, indicating that 3.3% of subjects reported zero intake of poultry or red meat over the two 24-hr periods.

### Vegetarians

**Figure 1:**
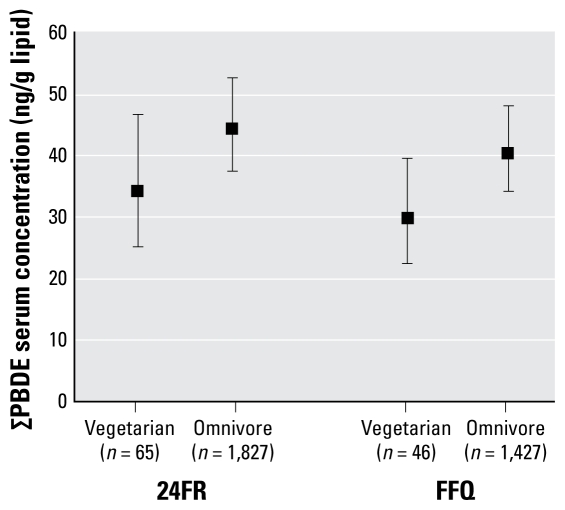
GM ∑PBDE concentrations in vegetarians and omnivores based on two distinct dietary assessments: 24FR and 1-year FFQ. Means are adjusted for race/ethnicity, sex, age, age^2^, PIR, and BMI. Error bars represent 95% CIs. For 24FR, *p*= 0.006; for FFQ, *p*= 0.009.

Figure 1 shows that vegetarians had significantly lower GM PBDE concentrations than did omnivores, as assessed by both the 24FR and FFQ: 34.1 versus 44.3 ng/g lipid (24FR) and 29.7 versus 40.4 ng/g lipid (FFQ). These differences were estimated while adjusting for sex, age, race/ethnicity, PIR, and BMI. In other words, vegetarians classified by either dietary assessment tool had 23–27% lower serum PBDE concentrations than did omnivores. Results were generally similar on a congener-specific basis, except that BDE-28 and BDE-153 were not significantly reduced in vegetarians as classified using the FFQ.

### Total fat intake

We first examined the association between serum PBDEs and total daily fat intake (uncategorized by food type) to assess whether food categorization was important in determining PBDE concentrations based on diet. In adjusted analyses, BDE-153 was the only congener significantly associated with total daily fat intake (log β = 0.0018; *p* = 0.048).

### Food categories

Table 3 presents the model results for poultry fat and red meat fat as determined by 24FR, adjusted for race/ethnicity, sex, age, PIR, and BMI. Poultry fat intake was a significant determinant of BDEs 28, 47, 99, 100, and 153 and ∑PBDE. Red meat fat intake was a significant determinant of BDE-100, BDE-153, and ∑PBDE. For both food types, ∑PBDE levels were lowest in the low-consumption groups, higher in the moderate-consumption groups, and highest in the high-consumption groups. These positive trends were also consistently observed for both poultry and red meat when serum PBDEs were analyzed on a congener- specific basis. With very few exceptions, serum PBDEs were not significantly associated with the other food categories (dairy, egg, seafood, non animal, or mixed/unclear) when analyzed on a congener-specific basis or as ∑PBDE [see Supplemental Material, Table 3 (doi:10.1289/ehp.0900817.S1)].

**Figure 2:**
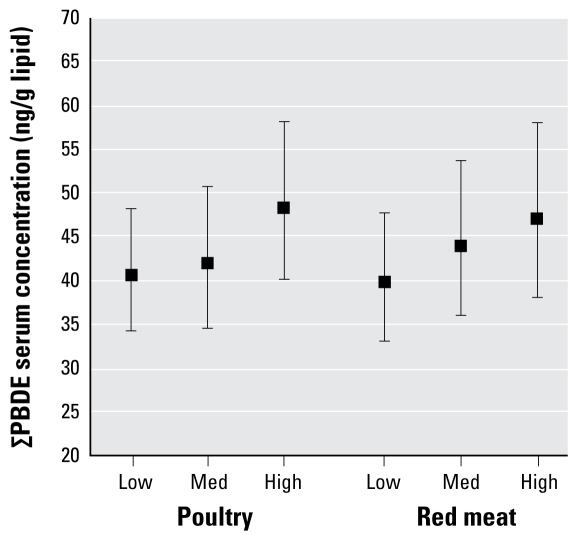
GM ∑PBDE concentrations by tertiles of mean poultry and red meat fat intake derived from a 24FR. Means are adjusted for race/ethnicity, sex, age, age^2^, PIR, and BMI. Error bars represent 95% CIs. Tests for trend: *p* = 0.0005 for poultry; *p* = 0.06 for red meat.

To facilitate interpretation, Figure 2 presents the adjusted GM concentrations of ∑PBDE predicted by poultry and red meat consumption as determined by 24FR. These estimates were generated using the model results presented in Table 3. For instance, the adjusted GM concentration of 48.3 ng/g lipid in the high poultry group is 19% higher than the adjusted GM concentration of 40.6 ng/g lipid in the low poultry group. Table 3 presents a parameter estimate of 0.17 (95% CI, 0.08–0) for this same comparison; exponentiation yields 1.19 (i.e., a 19% increase).

**Figure 3:**
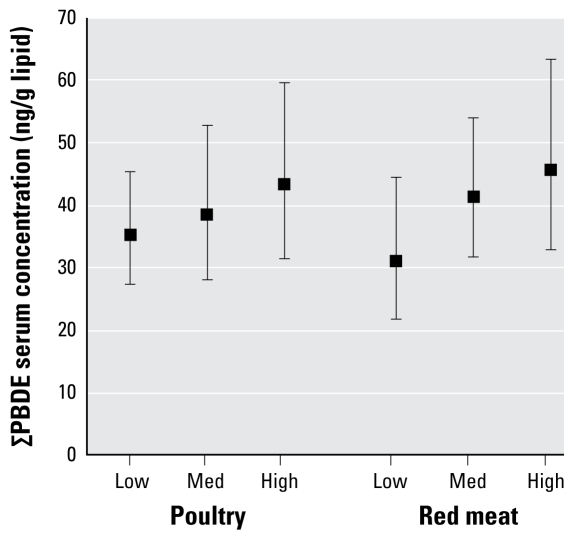
GM ∑PBDE concentrations by categories of mean poultry and red meat intake derived from an FFQ. Means adjusted for race/ethnicity, sex, age, age^2^, PIR, and BMI. Error bars represent 95% CIs. Tests for trend: *p* = 0.08 for poultry; *p* = 0.19 for red meat.

Supplemental Material, Table 4 (doi: 10.1289/ehp.0900817.S1), presents the model results for daily servings of poultry and red meat as determined by FFQ, adjusted for race/ethnicity, sex, age, PIR, and BMI. Figure 3 presents the adjusted GM serum concentrations of ∑PBDE corresponding to different categories of poultry and red meat consumption as determined by the FFQ. We observed consistent, but not significant, trends across tertiles of poultry and red meat for ∑PBDE and individual congeners, with two exceptions: We observed significant positive trends for red meat intake and BDE-153 and for poultry intake and BDE-28. We found no associations between PBDEs and dairy, seafood, egg, or non animal food intake.

## Discussion

Although previous studies have found PBDEs present in food products, the contribution of diet to overall PBDE exposure in North America is not well understood. Using 2003–2004 NHANES data, we found that consumption of poultry and red meat are significant determinants of PBDE body burdens. Based on the 24FR diet data, serum ∑PBDEs are 23% lower in vegetarians than in omnivores, 19% higher among heavy poultry consumers compared with light consumers (48.3 vs. 40.6 ng/g lipid), and 18% higher among heavy red meat consumers compared with light consumers (47.0 vs. 39.8 ng/g lipid).

Short of exhaustive methodologies that are rarely feasible (e.g., longitudinal duplicate diets), obtaining dietary information via questionnaire requires a compromise between the level of detail collected and breadth of coverage. Two of the most common methods, 24FRs and FFQs, represent opposite ends of this spectrum. Because of the long estimated half-lives of the PBDE congeners we investigated (Geyer et al. 2004), long-term dietary patterns should be more relevant than food intake measured over a few days. Although 24FRs are designed to provide accurate and detailed information on intake over a short period of time, they are not wholly representative of participants’ usual diets and would be expected to result in misclassification of exposure when long-term diet is the more appropriate exposure index. Under such circumstances, measurements of association can be considerably weakened, even undetectable (Willett 1998). Given the potential for nondifferential exposure misclassification, it is likely that the true relationship between diet and serum PBDE concentrations is even stronger than we have observed using 24FR.

Although the FFQ estimates food consumption over the previous year, it cannot provide the same accuracy of recall as the 24FR. Results from the FFQ were consistent with the 24FR findings but lacked the same level of statistical significance. One reason may be a key limitation of the NHANES FFQ: lack of portion size information. For example, consider the following question: “How often did you eat baked, broiled, roasted, stewed, or fried chicken (including nuggets)?” Without serving size information, the ability to compare responses between individuals is limited. Summing responses to different questions within food groups likely compounds the problem, increasing the potential for measurement error in overall intake estimates. Consequently, the FFQ data may lack the detail necessary to measure long-term diet accurately. Nevertheless, the FFQ data yielded statistically significant differences in serum PBDEs between vegetarians and omnivores as well as consistent, but not significant, associations between poultry and red meat and all five PBDE congeners. The consistency of our results using two completely different measures of food consumption suggests that diet—specifically poultry and red meat—is an important source of PBDEs in the U.S. general population. Using either method, our models employ consumption of food as a proxy for consumption of PBDEs in food, another source of exposure misclassification. The variation of PBDE concentrations within food groups (Huwe and Larsen 2005; Schecter et al. 2006b) is likely to result in underestimation of the importance of diet as a route of exposure (Wu et al. 2007).

Our findings are consistent with earlier exposure-factor studies estimating that poultry and red meat are important dietary sources of PBDEs in the United States (Schecter et al. 2006b) and that the contribution of PBDEs from poultry exceeds that from beef or pork (Huwe and Larsen 2005). Our findings are also consistent with those of Wu et al. (2007), who found that total meat intake (poultry plus red meat) was a significant determinant of penta-BDE congeners in breast milk collected from 46 first-time mothers in Massachusetts.

However, the lack of associations between PBDEs and either seafood or dairy is somewhat inconsistent with previous findings. Several studies of PBDEs in food have found the highest levels in fish and seafood (Domingo et al. 2008; Ohta et al. 2002; Schecter et al. 2008). One possible explanation for this apparent inconsistency is that seafood makes up a relatively small portion of most American diets, whereas poultry and red meat are consumed much more frequently (Huwe and Larsen 2005; Schecter et al. 2008). Similarly, Wu et al. (2007) found an association between PBDEs in breast milk and dairy fat intake. One possible explanation for this difference is the FFQ used by Wu et al. (2007) to measure longer-term diet. They also asked about serving sizes and used photographs to aid with size determination, a format that may have improved the ability to assess the role of dairy products in PBDE exposure.

Although results were similar across all congeners, BDE-153 was an occasional exception and was not as strongly correlated with the other PBDEs. These findings are consistent with previous studies that reported differences in congener profiles involving BDE-153. Although BDE-47 is the predominant congener in most people, several studies have identified subpopulations in which BDE-153 is the dominant congener (e.g., Eljarrat et al. 2005; Fängström et al. 2005; Ingelido et al. 2007; Johnson-Restrepo et al. 2005; Wu et al. 2007). In fact, 10.5% of participants in the present sample of the 2003–2004 NHANES were found to have higher levels of BDE-153 than BDE-47 (Sjödin et al. 2008). However, it is still unclear whether high BDE-153 concentrations are due to differences in exposure or toxicokinetics, or both. In our study, BDE-153 had a somewhat stronger association with red meat fat intake than did the other four congeners (Table 3). BDE-153 was also the only congener significantly associated with total daily fat intake.

Our examination of demographic factors and PBDEs produced results that are consistent with those of Sjödin et al. (2008). We found higher PBDEs in males and younger age groups, with some evidence for a curvilinear relationship with age. Whites tended to have lower serum PBDEs, but after adjusting for diet, BMI, PIR, age, and sex, most race/ethnicity differences disappeared. Results of the two demo graphic variables not examined by Sjödin et al. (2008), PIR and BMI, were particularly interesting. In crude analyses, we observed a striking trend of increasing PBDEs with increasing poverty. However, only BDE-100 remained significantly associated with PIR after adjusting for covariates (primarily age and race/ethnicity). PBDEs tended to increase with decreasing categories of BMI in crude analyses, but only BDE-153 remained significantly associated with BMI after adjusting for other covariates.

Strengths of our study include the large, representative sample provided by NHANES and the robust results using two distinct measures of diet. An important limitation in our analysis is the lack of data on other sources of exposure to PBDEs, particularly house dust. Some degree of confounding by dust exposure is possible, but this appears unlikely to explain the effect of diet after controlling for socio economic status and other demographic variables. In addition, our earlier work found that diet and PBDE dust concentrations were independent predictors of PBDE body burdens in first-time mothers from the Boston area (Wu et al. 2007). Using NHANES, Zota et al. (2008) recently reported differences in PBDE serum concentrations between California and the rest of the United States but did not examine diet. Future work should explore the combined effect of diet, dust, and geography. Although we have shown that diet is an important route of exposure for the general U.S. population, its contribution relative to environmental exposure thus remains uncertain. We were also unable to explore the connection between diet and BDE-209, for which data are not yet available in the 2003–2004 NHANES. Our findings, therefore, are limited to exposure to BDEs 28, 47, 99, 100, and 153, all present in the penta-BDE formulation.

## Conclusions

Our study offers the first large-scale look at the effect of the American diet on PBDE body burdens, showing significant associations with poultry and red meat consumption. PBDEs may enter the food chain in several ways, including contamination of food during processing or packaging and general contamination of the environment via emissions of PBDEs at various points of the life cycle of consumer products. As PBDE-containing products continue to degrade and enter the waste stream in larger amounts, future exposure to PBDEs may begin to shift more heavily from the indoor environment to the outdoor environment and, consequently, the diet (Harrad and Diamond 2006). This study highlights the need for research into the pathways of PBDEs into the food supply, particularly commercial animal products in the United States.

## Figures and Tables

**Table 1:** Demographic characteristics for subsets of the 2003–2004 NHANES [*n* (%)].

Categories	PBDE data set	24FR subset	FFQ subset
Total sample	2,040	1,971	1,536
Sex			
Female	1,046 (51)	1,007 (51)	829 (54)
Male	994 (49)	964 (49)	707 (46)
Race/ethnicity			
White	993 (49)	967 (49)	780 (51)
Black	492 (24)	474 (24)	363 (24)
Hispanic	555 (27)	530 (27)	393 (26)
Age (years)			
12–19	622 (30)	609 (31)	427 (28)
20–39	507 (25)	482 (24)	358 (23)
40–59	424 (21)	406 (21)	341 (22)
≥ 60	487 (24)	474 (24)	410 (27)
PIR			
< 1	472 (23)	456 (23)	334 (22)
1–2	502 (25)	487 (25)	364 (24)
2–4	505 (25)	485 (25)	389 (25)
≥ 4	452 (22)	443 (22)	369 (24)
Missing	109 (5)	100 (5)	80 (5)
BMI			
Underweight	102 (5)	98 (5)	70 (5)
Normal	761 (37)	736 (37)	558 (36)
Overweight	605 (30)	581 (29)	460 (30)
Obese	540 (26)	529 (27)	427 (28)
Missing	32 (2)	27 (1)	21 (1)
Poultry			
Low	NA	700 (36)	966 (63)
Medium	NA	610 (31)	417 (27)
High	NA	661 (34)	153 (10)
Red meat			
Low	NA	656 (33)	79 (5)
Medium	NA	658 (33)	1,121 (73)
High	NA	657 (33)	336 (22)

NA, not applicable.

**Table 2:** GM serum PBDE concentrations (ng/g lipid) for the 24FR subset of the 2003–2004 NHANES.

Category	BDE-28 (*n* = 1,921)	BDE-47 (*n* = 1,947)	BDE-99 (*n* = 1,918)	BDE-100 (*n* = 1,971)	BDE-153 (*n* = 1,970)	∑PBDE (*n* = 1,892)
Total sample	1.3	23.2	5.6	4.4	6.1	44.1

Sex						
Female	1.2	22.2	5.3	4.1	5.1	40.5
Male	1.3	24.2	5.9	4.7	7.4	48.2
*p*-Value^a^	0.07	0.04	0.003	0.01	< 0.0001	< 0.0001

Race/ethnicity						
Black	1.2	25.1	6.5	4.9	6.9	47.6
Hispanic	1.4	26.4	6.1	4.8	5.6	47.2
White	1.2	20.7	5.0	3.9	6.0	41.0
*p*-Value^a^	0.0007	0.007	0.02	0.06	0.04	0.15

Age (years)						
12–19	1.3	29.5	7.2	5.4	8.1	55.5
20–39	1.2	22.2	5.3	4.4	6.3	42.9
40–59	1.2	19.2	4.8	3.6	4.8	36.2
≥ 60	1.4	20.9	5.0	3.9	5.1	40.2
*p*-Value^a^	0.02	< 0.0001	< 0.0001	< 0.0001	< 0.0001	< 0.0001
Trend *p*-value	0.21	< 0.0001	< 0.0001	< 0.0001	< 0.0001	< 0.0001

PIR						
< 1	1.3	25.8	6.2	4.8	6.6	49.0
1–2	1.3	24.9	6.1	4.8	6.6	47.4
2–4	1.2	21.2	5.1	4.1	5.9	41.0
≥ 4	1.2	21.1	5.1	3.9	5.6	39.6
*p*-Value^a^	0.02	< 0.0001	0.0002	< 0.0001	0.21	0.0009
Trend *p*-value	0.08	0.006	0.02	0.001	0.09	0.004

BMI						
Underweight	1.3	29.3	7.5	6.3	14.9	66.8
Normal	1.2	23.5	5.9	4.4	7.3	45.8
Overweight	1.3	23.0	5.6	4.3	5.6	43.2
Obese	1.3	21.6	4.9	4.1	4.5	39.1
*p*-Value^a^	0.26	0.13	0.01	0.06	< 0.0001	0.009
Trend *p*-value	0.32	0.23	0.009	0.17	< 0.0001	0.02

a *p*-Values are for unadjusted associations between covariates and PBDEs using analysis of variance.

**Table 3:** Association of serum PBDEs with covariates and tertiles of poultry and red meat fat derived from 24FR [log β (*p*-value)].^a^

Category	BDE-28^b^	BDE-47^b^	BDE-99^b^	BDE-100^b^	BDE-153^b^	∑PBDE^b^
Poultry fat						
High	0.12 (0.02)	0.18 (0.002)	0.15 (0.01)	0.20 (0.0008)	0.17 (0.002)	0.17 (0.001)
Med	0.02 (0.67)	0.01 (0.85)	0.02 (0.68)	0.04 (0.57)	0.04 (0.51)	0.03 (0.58)
Low	Reference	Reference	Reference	Reference	Reference	Reference
Trend	(0.02)	(0.0005)	(0.006)	(0.0007)	(0.005)	(0.0005)
Red meat fat						
High	0.14 (0.07)	0.13 (0.10)	0.14 (0.06)	0.18 (0.03)	0.24 (0.005)	0.17 (0.0497)
Med	0.09 (0.10)	0.07 (0.35)	0.08 (0.25)	0.09 (0.28)	0.15 (0.06)	0.10 (0.20)
Low	Reference	Reference	Reference	Reference	Reference	Reference
Trend	(0.09)	(0.11)	(0.08)	(0.03)	(0.005)	(0.06)
Sex						
Female	−0.03 (0.34)	−0.06 (0.20)	−0.07 (0.05)	−0.08 (0.10)	−0.33 (< 0.0001)	−0.14 (0.0009)
Male	Reference	Reference	Reference	Reference	Reference	Reference
Race/ethnicity						
Black	−0.08 (0.55)	0.07 (0.52)	0.15 (0.14)	0.08 (0.46)	0.02 (0.82)	0.04 (0.72)
Hispanic	0.13 (0.07)	0.15 (0.08)	0.11 (0.16)	0.11 (0.26)	−0.18 (0.14)	0.05 (0.58)
White	Reference	Reference	Reference	Reference	Reference	Reference
Age (years)	−0.01 (0.006)	−0.03 (< 0.0001)	−0.03 (< 0.0001)	−0.02 (0.002)	−0.01 (0.01)	−0.02 (0.0001)
PIR						
< 1	0.09 (0.26)	0.05 (0.52)	0.05 (0.56)	0.09 (0.25)	0.14 (0.05)	0.11 (0.11)
1–2	0.08 (0.19)	0.07 (0.15)	0.08 (0.23)	0.14 (0.008)	0.17 (0.10)	0.12 (0.04)
2–4	−0.01 (0.88)	−0.07 (0.38)	−0.08 (0.37)	−0.02 (0.85)	0.04 (0.47)	−0.02 (0.77)
≥ 4	Reference	Reference	Reference	Reference	Reference	Reference
Trend	(0.20)	(0.20)	(0.37)	(0.02)	(0.13)	(0.08)
BMI						
Underweight	−0.01 (0.93)	0.07 (0.56)	0.22 (0.12)	0.26 (0.09)	1.06 (< 0.0001)	0.34 (0.02)
Normal	−0.09 (0.16)	−0.03 (0.67)	0.08 (0.21)	0.003 (0.96)	0.43 (< 0.0001)	0.07 (0.25)
Overweight	0.01 (0.85)	0.04 (0.58)	0.12 (0.12)	0.04 (0.52)	0.22 (0.02)	0.08 (0.26)
Obese	Reference	Reference	Reference	Reference	Reference	Reference
Trend	(0.19)	(0.76)	(0.17)	(0.89)	(< 0.0001)	(0.20)

a Adjusted for poultry, red meat, dairy, sex, race/ethnicity, age, age^2^, PIR, and BMI.

b Exponentiation of log β = multiplicative increase in PBDEs per unit change in predictor (e.g., log β = 0.12 is a 13% increase).
